# Association between smoking cessation and short-term health-care use: results from an international prospective cohort study (ATTEMPT)

**DOI:** 10.1111/add.12281

**Published:** 2013-08-14

**Authors:** Emma Beard, Lion Shahab, Susan J Curry, Robert West

**Affiliations:** 1 Cancer Research UK Health Behaviour Research Centre, Department of Epidemiology and Public Health, University College LondonLondon, UK; 2 Department of Health Management and Policy, College of Public Health, University of IowaIowa City, IA, USA

**Keywords:** Cigarettes, cost–benefit analysis, health-care costs, health-care use, hospitalization, smoking cessation

## Abstract

**Background and aims:**

Previous studies have found that smoking cessation is associated with a short-term increase in health-care use. This may be because ‘sicker’ smokers are more likely to stop smoking. The current study assessed the association between smoking cessation and health-care use, adjusting for pre-cessation physical and mental health conditions.

**Design/setting:**

Data came from the ATTEMPT cohort, a multi-national prospective survey of smokers in the United States, Canada, United Kingdom, France and Spain, that lasted 18 months (with follow-ups every 3 months).

**Participants:**

A total of 3645 smokers completed the baseline questionnaire. All participants smoked at least five cigarettes per day, intended to quit smoking within the next 3 months and were between 35 and 65 years of age.

**Measurements:**

Participants were asked questions about their socio-demographic and smoking characteristics, as well previous smoking-related morbidities. Participants were also asked to report their health-care use in the previous 3 months i.e. emergency room (ER) visits, hospitalization, whether hospitalization required surgery, and health-care appointments.

**Findings:**

A total of 8252, 4779 and 1954 baseline episodes of smoking were available for 3, 6 and 12 months, respectively. Of these, 2.8% (*n* = 230), 0.9% (*n* = 40) and 0.7% (*n* = 14) were followed by 3, 6 and 12 months of abstinence. No significant differences were found among 3, 6 or 12 months of abstinence and ER visits, hospitalization and whether hospitalization required surgery or health-care visits. However, 6-month smoking cessation episodes were associated with higher odds of reporting an appointment with a dietician.

**Conclusion:**

Smoking cessation does not appear to be associated with a substantial short-term increase or decrease in health-care use after adjusting for pre-cessation morbidities.

## Introduction

Clear health benefits accrue from quitting smoking. Smoking cessation lowers the risk of cancer, coronary heart disease, aortic aneurysm, stroke, peripheral vascular disease, chronic obstructive pulmonary disease (COPD) and mortality; it also leads to improved lung function and reduces the incidence of infertility 1. These health benefits are translated into long-term cost savings for health-care services 2,3. In the United States smoking-related morbidities account for 6–8% of national health-care spending 4, while in 2005–06 smoking-related conditions were estimated to have cost the National Health Service in England £5.2 billion 5.

Despite smoking cessation reducing use of health-care services in the long term, several studies have found an increase in use and health-care costs around the time of cessation 6–11. This may be because many smokers stop once they have become ill 12–14 but it is also possible that the immediate aftermath of quitting leads to an increase in health-care service use.

Quitting smoking might motivate smokers to adopt healthier life-styles, which may include seeking health-care advice. Alternatively, nicotine withdrawal symptoms, including weight gain and flu and cold-like symptoms in the initial weeks following abstinence, may motivate help-seeking behaviour 15,16. Thirdly, ex-smokers may be inclined to visit health-care professionals in order to reduce the risk of relapse, i.e. for further behavioural and/or medicinal support. Fourthly, it is possible that stopping smoking results in some kind of physiological disturbance which precipitates an illness episode. Finally, smokers who are in the early stages of an illness episode may be motivated to quit smoking and the natural history of the episode causes help-seeking once cessation has occurred.

Baumeister *et al*. 17 recently assessed the hypothesis that the association between smoking cessation and health-care use was due to sicker smokers attempting to quit, rather than smoking cessation precipitating health-care use. Baumeister *et al*. 17 used retrospective self-reported data from a sample of more than 4000 adults from Germany. After adjusting for health-care status and socio-economic variables, no association was found between time since smoking cessation and risk of hospitalization. However, the study used a retrospective cross-sectional design which may be subject to recall bias. In a recent prospective study of health-care use and costs among primary care patients receiving evidence-based smoking cessation treatment, Hockenberry *et al*. 8 concluded that smoking cessation therapy does not raise short-term health-care costs, and by the sixth quarter, post-quit sustained quitters were significantly less expensive than continuing smokers.

If quitting precipitates illness that requires health service care due to the psychological or physical effects of the cessation process, any cost–benefit analysis would need to take these additional costs into account. Additional prospective studies of the association between smoking cessation and short-term health-care use are therefore needed, adjusting for a range of socio-demographic and smoking characteristics, as well as psychological and physical morbidities. The current study, using data from the ATTEMPT cohort, aimed to explore the association prospectively between smoking cessation and a full range of health services, including emergency room (ER) visits, hospitalization and health-care appointments, and to assess the reasons for using these services.

## Methods

Data for this study come from the ATTEMPT cohort, a multi-national prospective study of smokers which collects data on smoking and socio-demographic characteristics, as well as physical and mental health outcomes. Participants were recruited by Harris Interactive, Inc., which maintains a market research panel of several million panellists in more than 125 countries. Each panel member volunteers to complete regular online surveys for research purposes in exchange for points that can be redeemed against merchandise. Full details of the study methodology are provided elsewhere 18. Only those panellists who smoked at least five cigarettes per day, intended to quit within the following 3 months and were aged between 35 and 65 years were included into the current study. The sample was restricted to those who expressed a desire to quit so that the chances of obtaining an adequate number of quit attempts during the follow-up period was maximized. Both light and moderate-to-heavy smokers were included so that the broad range of smoking habits were captured; the age range of 35–65 was chosen so that the sample included people who were likely to be beginning to experience health problems associated with smoking but before there would be substantial confounding due to loss of smokers because of mortality. Panellists were restricted to United States, Canada, the United Kingdom, France and Spain (phase 2 of data collection). A total of 3645 panellists were eligible to participate. Participants completed self-report questionnaires at baseline and follow-up (i.e. 3, 6, 9, 12, 15 and 18 months).

## Measures

### Demographic characteristics and pre-morbidity

Gender, age, employment status, marital status, body mass index (BMI), Fagerström Test of Nicotine Dependence (FTND), ethnicity and country were assessed at baseline and at subsequent follow-up surveys. Diagnosis of previous smoking related-morbidity was also assessed. Smokers were asked ‘Have you ever been told by a doctor or other health care professional that you had any of the following conditions?’ (heart or circulation condition; respiratory condition; endocrine, hormone or metabolic condition; digestive condition; nervous system condition; cancer; ear, nose or throat condition; bone, joint or muscle condition; skin condition; reproductive or urinary condition; other condition).

### Smoking status

At each follow-up point smokers were asked: ‘Are you currently a cigarette smoker?’ (yes, no) and ‘How many days has it been since you last smoked?’. Those who reported that they had quit smoking and had not smoked in the previous 90 days were classified as being abstinent for 3 months, while those who reported that they had not quit smoking and had smoked in the previous 90 days were classified as continuing smokers. Those who were abstinent for more than 7 days, but fewer than 3 months, and those who had been abstinent for more than 3 months, were excluded from the analysis. [Note that the percentage of participants classified as having made a 3-month quit episode should not be interpreted as a relapse rate at 3 months, as the comparison is between continuing smokers as opposed to those who attempted to quit and failed, i.e. were abstinent for fewer than 3 months].

These classifications were corroborated by asking smokers three further questions: (i) ‘Just to confirm, in the last 3 months (90 days) have you smoked any cigarettes (even a puff) (yes, no); (ii) Have you smoked any cigarettes today (yes, no); and (iii) During the past 3 months (90 days), what is the longest period of time you stopped smoking cigarettes because you were trying to quit smoking for good?’. Those classified as being abstinent for 3 months were re-classified if they reported having smoked ‘cigarettes today’ and that their longest period of abstinence was less than 3 months. However, in all cases a 7-day leeway was given for classification, i.e. 3-month quitters had to be abstinent on the day of testing and to have quit for between 83 and 90 days. This 7-day leeway ensured that those who had quit smoking for close to 3 months were not excluded from the analysis.

Similar categorizations were used for smokers who had been abstinent for 6 months and 12 months. For example, those who reported that they had quit smoking and had not smoked in the previous 180 days were classified as being abstinent for 6 months, while those who reported that they had quit smoking and had not smoked in the previous 360 days were classified as being abstinent for 12 months. Each participant could contribute multiple ‘abstinent’ or ‘smoking’ episodes during the 18-month period.

### Health-care use

Finally, participants were asked about their health-care use. This focused on three main areas: (i) visits to ER; (ii) hospitalization and (iii) health-care appointments.

#### Visits to ER

Participants were asked: ‘During the past 3 months (90 days), how many times have you gone to a hospital emergency room about your own health’ and ‘What was the primary reason for your visit to the emergency room (ER)?’ (heart or circulation condition; respiratory condition; endocrine, hormone or metabolic condition; digestive condition; nervous system condition; cancer; ear, nose or throat condition; bone, joint or muscle condition; other condition). ER refers to emergency departments, casualty departments and accident and emergency.

#### Hospitalization

Participants were asked: ‘During the past 3 months (90 days), how many times have you been hospitalized’ and ‘What was the primary reason (e.g. medical condition) for your hospitalization?’ (heart or circulation condition; respiratory condition; endocrine, hormone or metabolic condition; digestive condition; nervous system condition; cancer; bone, joint or muscle condition; other condition). Participants were also asked: ‘During which hospitalization, if any, did you have any surgery performed?’

#### Health-care appointments

Participants were asked: ‘Please select each health care service for which you have had any appointments in the past 3 months (90 days)’ [out-patient surgery; out-patient diagnostic procedure (for example, computerized tomography scan, magnetic resonance imaging, biopsy, endoscopy and colonoscopy); laboratory test (for example, blood or urine test); treatment administration (for example, dialysis, blood transfusion, radiation, chemotherapy, injection, infusion); general practitioner (also includes family physician, internist and primary care physician consultation); specialist smoking cessation consultation; other specialist physician consultation (for example, cardiologist, pulmonologist or gynaecologist); psychological/mental health counselling; nurse consultation; dietician consultation; physical therapy; chiropractor visit; acupressure/acupuncture; homeopathy; other health-care service provider].

## Analysis

Data were analysed using SPSS version 20.0 (IBM, New York, NY, USA). In unadjusted analyses, between-group differences [(i) those followed-up versus those not followed-up at 3 months (6 and 12 months) and (ii) those who were abstinent at the 3-month (6-month and 12-month) follow-up versus continuing smokers] were assessed with χ^2^ test and *t*-test analyses for categorical and continuous variables, respectively. As smokers could contribute multiple episodes for the analysis between abstinence and health-care use, generalized estimating equations (GEE) were used. GEE is a method for handing correlated discrete data that would typically be analysed using traditional generalized linear models (GLM). The primary difference from traditional GLM is its ability to account for the within-subject covariance structure for the response data. The fitted model is specified through a link function (which relates the mean response to the regression equation), an assumed distribution for the response (e.g. binomial or Poisson) and a working correlation matrix. Common link functions are the logit function *g*(*x*) = log[*x*(1–*x*)] for binary responses and the log function *g*(*x*) = log(*x*) for Poisson counts. There are a number of options for the type of correlation between observations, e.g. (i) exchangeable: which should be adopted when any two observations within a cluster are equally correlated but there are no correlations between observations from different clusters; (ii) autoregressive: should be used when repeated measures are correlated most strongly when close together in time and correlated least when furthest apart in time; and (iii) unstructured: places no constraints on correlations.

In the current study, GEE analyses were based on the logit link function and used an exchangeable correlation matrix to model the interdependence between data resulting from some participants reporting multiple abstinence and/or continuous smoking episodes. Analyses adjusted for age, gender, employment status, marital status, ethnicity, BMI, FTND and smoking-related morbidity. In order to control for unidentified country specific confounders, a categorical variable specifying each individual country was also included in the analyses. Sensitivity analyses were conducted by assessing the association between health-care use and abstinence during each follow-up period using multiple-logistic regression. GEEs were also used to assess whether differences existed between 3- and 6-month abstinence episodes and smoking continuation episodes in terms of the type of health-care appointment that was attended and whether hospitalization required surgery; 95% confidence intervals (CI) are given unless stated otherwise. Where complete separation was evident, i.e. the presence of one or more covariates perfectly predicted the outcome of interest, the variables were deleted from the adjusted analysis. If complete separation was due to the main predictor variable (i.e. smoking status: abstinence versus smoking continuation) the analysis was terminated. Differences were not assessed between the reasons for ER visits and hospitalization due to the small sample size; instead descriptive statistics are presented.

## Results

### Participants

A total of 68.2% (*n* = 2485) smokers completed the 3-month follow-up questionnaire, 59.7% (*n* = 2175) completed the 6-month follow-up questionnaire, 54.2% (*n* = 1975) completed the 9-month follow-up questionnaire, 45.0% (*n* = 1640) the 12-month follow-up questionnaire, 33.8% (*n* = 1233) the 15-month follow-up questionnaire and 31.8% (*n* = 1160) the 18-month follow-up questionnaire (see Supporting information and Supplementary Table S1 for comparisons between those followed and not followed-up).

Of those followed-up at 3 months, 8.3% (CI = 7.2–9.4) reported that they were abstinent for 3 months at least once during the 18-month period of follow-up, while 1.8% (CI = 1.4–2.5) of those followed-up at 6 months reported that they were abstinent for 6 months during the study period. Finally, 0.9% (CI = 0.5–1.4) of those followed-up at 12 months reported that they were abstinent for at least 12 months. Among those reporting abstinence for 3 months, 88.3% (*n* = 181; CI = 83.2–92.0) had been abstinent only once during the 18-month period, 11.2% (*n* = 23; CI 7.6–16.3) twice, and 0.5% (*n* = 1; CI = 0.1–2.7) three times. In contrast, smokers who were abstinent for 6 or 12 months reported just one episode of abstinence. Those who had been abstinent at least once for 3 months differed from those who had not been abstinent for 3 months; they were more likely to have been diagnosed with an endocrine, hormone or metabolic disorder (χ^2^ = 3.83, d.f. 1, *P* = 0.050), on average had a significantly lower FTND score (*t* = 4.22, d.f. 2434, *P* < 0.001) and higher body mass index (*t* = −2.65, d.f. 2483, *P* = 0.008). Those who had been abstinent for 6 and 12 months did not differ from those who had not been abstinent for 6 or 12 months (see Table 1).

**Table 1:** Baseline sample characteristics by smoking status

	Followed-up at 3 months: reported 3 months abstinence over the 18-month period		Followed-up at 6 months: reported 6 months abstinence over the 18-month period		Followed-up at 12 months: reported 12 months abstinence over the 18-month period	
	Yes (n *=* 205)	No (n *=* 2280)	Yes (n *=* 40)	No (n *=* 2135)	Yes (n *=* 14)	No (n *=* 1626)
Socio-demographic characteristics						
Mean (SD) age	46.7 (7.93)	45.9 (7.32)	47.0 (7.39)	46.0 (7.42)	47.3 (6.34)	46.3 (7.48)
% (*n*) Women	46.3 (95)	45.8 (1044)	40.0 (16)	45.8 (977)	42.9 (6)	45.2 (735)
% (*n*) Married^k^	52.2 (107)	48.9 (1110)	57.5 (23)	49.6 (1056)	71.4 (10)	51.5 (829)
% (*n*) Employed^l^	77.1 (158)	72.0 (1641)	85.0 (34)	72.7 (1553)	85.7 (12)	73.4 (1193)
% (*n*) Country						
United States	23.9 (49)	19.6 (446)	25.0 (10)	22.0 (469)	25.5 (363)	26.4 (430)
Canada	3.4 (7)	2.9 (65)	0.0 (0)	3.2 (68)	3.3 (47)	3.5 (57)
United Kingdom	31.2 (64)	29.9 (681)	32.5 (13)	27.9 (596)	26.2 (374)	26.4 (430)
France	27.3 (56)	35.8 (816)	32.5 (13)	34.4 (735)	32.1 (458)	31.1 (506)
Spain	14.1 (29)	11.9 (272)	10.0 (4)	12.5 (267)	12.9 (184)	12.5 (203)
% (*n*) White ethnicity	94.1 (193)	93.6 (2135)	90.0 (36)	93.3 (1991)	92.9 (13)	92.7 (1508)
Cigarette smoking characteristics						
Mean (SD) FTND score^m^	4.0 (2.44)	4.7 (2.40)***	4.2 (2.94)	4.6 (2.36)	5.6 (2.82)	4.7 (2.40)
Physical/mental health characteristics						
Mean (SD) body mass index	27.8 (5.49)	26.7 (5.57)**	28.0 (5.22)	26.9 (5.58)	28.2 (4.82)	27.1 (5.45)
% (*n*) Heart or circulation condition^a^,^n^	31.7 (65)	38.5 (878)	30.0 (12)	39.0 (832)	14.3 (2)	37.9 (617)
% (*n*) Respiratory condition^b^,^n^	24.9 (51)	24.6 (560)	30.0 (12)	23.6 (504)	28.6 (4)	23.4 (381)
% (*n*) Endocrine, hormone or metabolic disorder^c^,^n^	25.4 (52)	19.6 (448)*	27.5 (11)	20.7 (441)	28.6 (4)	21.1 (343)
% (*n*) Digestive condition^d^,^n^	19.0 (39)	17.3 (395)	17.5 (7)	17.1 (366)	35.7 (5)	17.1 (278)
% (*n*) Nervous system condition^e^,^n^	39.0 (80)	38.5 (877)	37.5 (15)	38.0 (812)	35.7 (5)	37.5 (610)
% (*n*) Cancer^f^,^n^	3.9 (8)	4.3 (97)	5.0 (2)	4.2 (89)	0.0 (0)	3.8 (61)
% (*n*) Ear, nose or throat condition^g^,^n^	35.6 (73)	34.4 (784)	35.0 (14)	34.3 (732)	42.9 (6)	35.1 (571)
% (*n*) Bone, joint or muscle condition^h^,^n^	35.6 (73)	31.7 (722)	32.5 (13)	32.1 (686)	28.6 (4)	32.5 (528)
% (*n*) Skin condition^i^,^n^	17.6 (36)	18.2 (415)	17.5 (7)	18.9 (403)	14.3 (2)	18.3 (298)
% (*n*) Reproductive or urinary condition^j^,^n^	13.2 (27)	16.1 (368)	17.5 (7)	16.0 (341)	21.4 (3)	16.6 (270)
% (*n*) Other^n^	13.7 (28)	12.6 (288)	7.5 (3)	12.9 (276)	0.0 (0)	13.2 (214)

* *P* < 0.05

** *P* < 0.01

*** *P* < 0.001.

a Includes (1) angina pectoris, (2) venous insufficiency, (3) peripheral artery disease, (4) heart attack, heart disease or other heart condition, (5) hypertension, (6) high cholesterol or abnormal triglycerides, (7) stroke.

b Includes (1) asthma, (2) acute bronchitis, (3) chronic obstructive pulmonary disease (COPD), (4) other respiratory condition.

c Includes (1) diabetes, (2) obesity.

d Includes (1) irritable bowel syndrome, Crohn's disease or ulcerative colitis, (2) stomach, duodenal or peptic ulcer, (3) hepatitis or cirrhosis.

e Includes (1) depression, (2) anxiety, (3) multiple sclerosis.

f Includes (1) cancer of the lung, throat, mouth, pancreas, bladder, uterus, oesophagus or kidney, (2) cancer of some other type.

g Includes (1) hay fever, allergic rhinitis, seasonal allergies, sinusitis, (2) chronic sinus infection, (3) chronic laryngitis or pharyngitis, (4) gum disease.

h Includes (1) osteoporosis, (2) arthritis, gout, lupus or fibromyalgia, (3) chronic back pain, (4) chronic joint pain, (5) chronic muscle pain, (6) other chronic pain.

i Includes (1) wrinkles or skin ageing, (2) other skin condition.

j Includes (1) sexual dysfunction, (2) menopause, (3) kidney or renal failure

k 11 missing

l nine missing

m 49 missing

n 18 missing. FTND = Fagerström Test of Nicotine Dependence; SD = standard deviation.

### Smoking episodes

For the analysis of 3-month abstinence, participants contributed 10 668 episodes of a possible 21 870 (if all had completed each follow-up point). Around 2500 episodes were excluded from the analysis of 3-month quit attempts, as they involved either an attempt to stop smoking in the previous 3 months which lasted between 8 and 82 days or an episode of abstinence which had lasted more than 3 months. This resulted in a final sample of 8252 episodes, of which 230 (2.8%) were classified as 3-month quit attempts and 8022 (97.2%) as smoking continuation. For the analysis of 6-month abstinence, participants contributed 8183 episodes of a possible 18 225 episodes. A further 4729 episodes were excluded from the 6-month analysis, as they involved either an attempt to stop smoking in the previous 6 months which lasted between 8 and 172 days or an episode of abstinence which had lasted more than 6 months. This resulted in a final sample of 4779 episodes, of which 40 (0.9%) were classified as a 6-month quit attempt while 4738 (99.1%) as smoking continuation. Finally, for the analysis of 12 months abstinence, participants contributed 4033 episodes of a possible 10 935 episodes, of which 2079 were excluded as they involved either an attempt to stop smoking in the previous 12 months which lasted between 8 and 357 days or an episode of abstinence which had lasted more than 12 months. This resulted in a final sample of 1954 episodes. Fourteen of these (0.7%) were classified as a 12-month quit attempt and 1940 (99.3%) as smoking continuation.

### Association with health-care use

Table 2 shows the percentage of episodes which involved at least one ER visit, at least one hospitalization and at least one health-care appointment in the 3 months following smoking cessation, as a function of the cohorts of interest. ER visits and hospitalization were mainly for ‘heart or circulation conditions’ and ‘other conditions’. Seeking help for bone, joint and muscle conditions was also common among smoking continuation episodes. The most common health-service appointments were for general practitioners and laboratory tests. Table 3 shows the results of the GEE analyses of these data. No significant differences in terms of ER visits, hospitalization or health-care visits were found between episodes involving abstinence (for either 3, 6 or 12 months) and episodes involving continued smoking. This was corroborated in the sensitivity analysis. There was only one significant difference in terms of the type of appointment attended: 6-month abstinence episodes were significantly more likely to be followed by a visit to a dietician compared to appointment visits during continuing smoking episodes [odds ratio (OR) = 8.18, CI = 2.00–33.55, *P* < 0.01]. No difference was found in terms of whether or not surgery was necessary during hospitalization.

**Table 2:** Health-care use at follow-up by smoking episode

	3-month quit episode		6-month quit episode		12-month quit episode	
	Yes (n *=* 230)	No (n *=* 8022)	Yes (n *=* 40)	No (n *=* 4738)	Yes (n *=* 14)	No (n *=* 1940)
% (*n*) Emergency room visit previous 3 months	7.4 (17)	5.6 (453)	9.8 (4)	5.5 (259)	14.3 (2)	5.1 (99)
Reason for emergency room visit^p^,^r^						
% (*n*) Heart or circulation condition^b^	11.8 (2)	10.4 (47)	25.0 (1)	8.5 (22)	0.0 (0)	10.1 (10)
% (*n*) Respiratory condition^c^	5.9 (1)	6.8 (31)	0.0 (0)	7.3 (19)	0.0 (0)	1.0 (1)
% (*n*) Endocrine, hormone or metabolic disorder^d^	0.0 (0)	1.5 (7)	0.0 (0)	1.2 (3)	0.0 (0)	1.0 (1)
% (*n*) Digestive condition^e^	0.0 (0)	4.4 (20)	0.0 (0)	1.9 (5)	0.0 (0)	3.0 (3)
% (*n*) Nervous system condition^f^	0.0 (0)	6.0 (27)	0.0 (0)	7.7 (20)	0.0 (0)	13.1 (13)
% (*n*) Cancer^g^	0.0 (0)	1.1 (5)	0.0 (0)	1.9 (5)	0.0 (0)	5.1 (5)
% (*n*) Ear, nose or throat condition^h^	0.0 (0)	5.7 (26)	0.0 (0)	5.8 (15)	0.0 (0)	7.1 (7)
% (*n*) Bone, joint or muscle condition^i^	0.0 (0)	14.3 (65)	0.0 (0)	17.0 (44)	0.0 (0)	17.2 (17)
% (*n*) Other^j^	58.8 (10)	80.6 (365)	75.0 (3)	66.8 (173)	100 (2)	64.6 (64)
% (*n*) Hospitalization previous 3 months	5.7 (13)	4.2 (333)	4.9 (2)	4.2 (201)	7.1 (1)	4.4 (86)
% (*n*) Hospitalization required surgery	46.2 (6)	52.0 (173)	0.0 (0)	51.7 (104)	0.0 (0)	48.8 (42)
Reason for hospitalization^q^,^r^						
% (*n*) Heart or circulation condition^b^	23.1 (3)	16.2 (54)	50.0 (1)	17.4 (35)	0.0 (0)	17.4 (15)
% (*n*) Respiratory condition^c^	15.4 (2)	6.9 (23)	0.0 (0)	3.5 (7)	0.0 (0)	0.0 (0)
% (*n*) Endocrine, hormone or metabolic disorder^d^	0.0 (0)	2.4 (8)	0.0 (0)	2.5 (5)	0.0 (0)	2.3 (2)
% (*n*) Digestive condition^e^	0.0 (0)	7.2 (24)	0.0 (0)	6.0 (12)	0.0 (0)	9.3 (8)
% (*n*) Nervous system condition^f^	0.0 (0)	4.2 (14)	0.0 (0)	6.0 (12)	0.0 (0)	8.1 (7)
% (*n*) Cancer^g^	30.8 (4)	6.9 (23)	0.0 (0)	4.5 (9)	0.0 (0)	1.2 (1)
% (*n*) Ear, nose or throat condition^h^	0.0 (0)	3.0 (10)	0.0 (0)	2.5 (5)	0.0 (0)	1.2 (1)
% (*n*) Bone, joint or muscle condition^i^	0.0 (0)	8.4 (28)	0.0 (0)	11.9 (24)	0.0 (0)	18.6 (16)
% (*n*) Other^j^	61.5 (8)	67.3 (224)	50.0 (1)	65.7 (132)	100 (2)	72.1 (62)
% (*n*) Health-care service appointment previous 3 months	59.1 (136)	55.2 (4426)	61.0 (25)	55.2 (2614)	64.3 (9)	55.9 (1084)
Reason for health-care service appointment^a^						
% (*n*) Out-patient surgery	7.4 (10)	7.7 (343)	4.0 (1)	7.7 (202)	11.1 (1)	7.1 (77)
% (*n*) Out-patient diagnostic procedure^k^	13.2 (18)	13.2 (584)	0.0 (0)	13.2 (345)	0.0 (0)	14.1 (153)
% (*n*) Laboratory test^l^	38.2 (52)	41.3 (1826)	48.0 (12)	42.3 (1106)	44.4 (4)	42.2 (457)
% (*n*) Treatment administration^m^	4.4 (6)	2.9 (127)	0.0 (0)	2.8 (74)	0.0 (0)	2.7 (29)
% (*n*) General practitioner^n^	70.6 (96)	69.9 (3095)	72.0 (18)	69.0 (1804)	77.8 (7)	70.5 (764)
% (*n*) Specialist smoking cessation consultation	3.7 (5)	2.4 (106)	0.0 (0)	1.6 (42)	0.0 (0)	1.1 (12)
% (*n*) Other specialist physician consultation^o^	20.6 (28)	24.0 (1064)	16.0 (4)	25.4 (665)	0.0 (0)	25.7 (279)
% (*n*) Psychological/mental health counselling	7.4 (10)	8.4 (371)	4.0 (1)	8.8 (231)	0.0 (0)	8.9 (96)
% (*n*) Nurse consultation	11.8 (16)	6.9 (304)	8.0 (2)	6.8 (117)	0.0 (0)	6.6 (72)
% (*n*) Dietician consultation	5.1 (7)	2.2 (97)	12.0 (3)	1.7 (44)	0.0 (0)	1.8 (20)
% (*n*) Physical therapy	7.4 (10)	7.5 (330)	4.0 (1)	8.0 (208)	11.1 (1)	6.7 (72)
% (*n*) Chiropractor visit	5.1 (7)	5.9 (255)	4.0 (1)	5.6 (147)	0.0 (0)	5.6 (61)
% (*n*) Acupressure/acupuncture	1.5 (2)	1.4 (62)	4.0 (1)	1.3 (35)	11.1 (1)	1.3 (14)
% (*n*) Homeopathy	0.7 (1)	1.4 (60)	4.0 (1)	1.2 (31)	0.0 (0)	1.1 (12)
% (*n*) Other health-care service provider	6.6 (9)	9.5 (419)	12.0 (3)	9.9 (258)	22.2 (2)	10.0 (108)

a Participants could choose more than one reason.

b Includes (1) angina pectoris, (2) venous insufficiency, (3) peripheral artery disease, (4) heart attack, heart disease or other heart condition, (5) hypertension, (6) high cholesterol or abnormal triglycerides, (7) stroke.

c Includes (1) asthma (2) acute bronchitis, (3) chronic obstructive pulmonary disease (COPD), (4) other respiratory condition.

d Includes (1) diabetes, (2) obesity.

e Includes (1) irritable bowel syndrome, Crohn's disease or ulcerative colitis, (2) stomach, duodenal or peptic ulcer.

f Includes (1) depression, (2) anxiety.

g Includes (1) cancer of the lung, throat, mouth, pancreas, bladder, uterus, oesophagus or kidney, (2) cancer of some other type.

h Includes (1) hay fever, allergic rhinitis, seasonal allergies, sinusitis, (2) chronic sinus infection, (3) chronic laryngitis or pharyngitis, (4) gum disease, (5) common cold, (6) influenza.

i Includes (1) chronic back pain, (2) chronic joint pain, (3) chronic muscle pain. (4)

j Includes trauma or serious accident.

k For example, computerized tomography (CT) scan, magnetic resonance imaging (MRI), biopsy, endoscopy and colonoscopy.

l For example, blood or urine test.

m For example, dialysis, blood transfusion, radiation, chemotherapy, injection, infusion.

n Also includes family physician, internist and primary care physician consultation.

o For example, cardiologist, pulmonologist or gynaecologist

p 80 missing

q 164 missing.

r Participants could report multiple reasons if they attended the emergency room (ER) or were hospitalized more than once.

**Table 3:** Association between quit attempts and health-care use

	Unadjusted			Adjusted^a^		
	3-month quit episodes versus smoking continuation episodes	6-month quit episodes versus smoking continuation episodes	12-month quit episodes versus smoking continuation episodes	3-month quit episodes versus smoking continuation episodes	6-month quit episodes versus smoking continuation episodes	12-month quit episodes versus smoking continuation episodes
	OR (95% CI)	OR (95% CI)	OR (95% CI)	(OR 95% CI)	OR (95% CI)	OR (95% CI)
% (*n*) Emergency room visit	1.16 (0.66–2.02)	1.59 (0.56–4.51)	3.03 (0.67–13.77)	1.14 (0.64–2.05)	1.73 (0.66–4.54)	2.45 (0.65–9.22)
% (*n*) Hospitalization	1.43 (0.80–2.54)	1.37 (0.38–4.94)	1.67 (0.22–13.00)	1.53 (0.83–2.81)	1.60 (0.42–6.19)	1.71 (0.24–12.05)
% (*n*) Hospitalization required surgery	0.65 (0.24–1.75)	e	d	0.45 (0.16–1.30)	e	d
% (*n*) Health-care service appointment	1.04 (0.81–1.32)	1.24 (0.73–2.08)	1.41 (0.47–4.24)	1.04 (0.78–1.39)	1.40 (0.77–2.53)	1.72 (0.53–5.59)
Reason for health-care service appointment						
% (*n*) Out-patient surgery	0.87 (0.41–1.83)	0.37 (0.2–6.01)	d	1.00 (0.46–2.18)	0.48 (0.03–6.74)	d
% (*n*) Out-patient diagnostic procedure^f^	0.92 (0.51–1.65)	e	d	0.99 (0.54–1.82)	e	d
% (*n*) Laboratory test^g^	0.88 (0.60–1.27)	1.19 (0.57–2.52)	d	0.94 (0.62–1.44)	1.35 (0.61–2.95)	d
% (*n*) Treatment administration^h^	1.54 (0.59–4.05)	e	d	1.60 (0.57–4.50)	e	d
% (*n*) General practitioner^i^	1.00 (0.70–1.44)	1.19 (0.52–2.70)	d	1.02 (0.69–1.50)	1.27 (0.51–3.17)	d
% (*n*) Specialist smoking cessation consultation	1.27 (0.45–3.59)	e	d	1.50 (0.41–5.52)	e	d
% (*n*) Other specialist physician consultation	0.71 (0.46–1.09)	0.66 (0.30–1.48)	d	0.81 (0.52–1.27)	0.77 (0.34–1.78)	d
% (*n*) Psychological/mental health counselling	1.00 (0.52–1.92)	1.69 (0.72–4.01)	d	1.08 (0.51–2.27)	0.99 (0.28–3.58)	d
% (*n*) Nurse consultation	1.33 (0.75–2.39)	1.38 (0.43–4.45)	d	1.26 (0.64–2.45)	1.45 (0.53–3.97)	d
% (*n*) Dietician consultation	1.66 (0.66–4.18)	5.91 (1.21–29.00)*	d	1.39 (0.59–3.27)	8.18 (2.00–33.55)**	d
% (*n*) Physical therapy	1.04 (0.57–1.93)	0.39 (0.3–4.72)	d	1.00 (0.55–1.83)	0.55 (0.06–3.39)	d
% (*n*) Chiropractor visit	1.08 (0.50–2.31)	0.92 (0.25–3.38)	d	1.14 (0.58–2.26)	0.88 (0.19–4.09)	d
% (*n*) Acupressure/acupuncture	0.64 (0.04–11.20)	1.64 (0.01–818.75)	d	1.06 (0.13–8.70)^b^	1.95 (0.004–140 573.411)^c^	d
% (*n*) Homeopathy	0.43 (0.04–5.31)	1.39 (0.01–297.11)	d	0.53 (0.04–7.30)^b^	2.36 (0.05–122.36)^b^	d
% (*n*) Other health-care service provider	0.82 (0.44–1.52)	1.33 (0.42–4.22)	d	0.92 (0.47–1.79)	1.36 (0.39–4.73)	d

* *P* < 0.05

** *P* < 0.01.

a Adjusted for Fagerström Test of Nicotine Dependence (FTND), age, ethnicity gender, marital status, employment status, country, body mass index (BMI) and pre-diagnosed physical and mental health conditions (i.e. heart or circulation condition; respiratory condition; endocrine, hormone or metabolic disorder; digestive condition; nervous system condition; cancer; ear, nose or throat condition; bone, joint or muscle condition; skin condition; reproductive or urinary condition; and other condition).

b Not adjusted for country due to quasi (complete) separation.

c Not adjusted for country or cancer due to quasi (complete) separation.

d Analysis not undertaken due to small sample size.

e Analysis not undertaken due to complete separation in the main predictor variable (smoking status).

f For example, computerized tomography (CT) scan, magnetic resonance imaging (MRI), biopsy, endoscopy and colonoscopy.

g For example, blood or urine test.

h For example, dialysis, blood transfusion, radiation, chemotherapy, injection and infusion.

i Also includes family physician, internist and primary care physician consultation. For example, cardiologist, pulmonologist or gynecologist. OR = odds ratio; CI = confidence interval.

## Discussion

This study investigated the relationship between smoking cessation and health-care use in the following months. No association was found between smoking cessation and subsequent ER visits, hospitalization or health-care service appointments. There was also no difference in terms of whether or not hospitalization required surgery; however 6-month smoking cessation episodes were associated with higher odds of reporting an appointment with a dietician.

In line with previous studies 11,17, our findings suggest that there is no initial increase in health-care use within 3 months following smoking cessation, as measured by ER visits, hospitalization and appointments with health-care services. Our study was also well powered to detect medium-sized changes in health-care use at 6 months, but clearly underpowered to draw firm conclusions about health-care use within 12 months. Meta-analyses are required which combine the longer-term data reported here with data from other studies, in order to determine the effects of long-term smoking cessation on hospital visits and other health-care use.

To our knowledge, this is the first prospective study to assess the association between smoking cessation and various health-care services which has also adjusted for multiple socio-demographic and smoking characteristics, as well as psychological and physical health complaints—all of which are important determinants of health-care use 19–22. We also adjusted for participants' country of residence, as differences in country health-care structure may impact upon use of services following abstinence, e.g. there are significant differences in the privatization of health-care in the United States and United Kingdom which could result in lower use. Unlike previous studies, the current study used a multi-country design and assessed the association between cessation and multiple health complaints and types of health-care services.

It appeared that recent ex-smokers did not seek help for conditions different from continuing smokers in the short term. The most common complaints involved ‘heart or circulation conditions’ and ‘other conditions’, e.g. trauma. However, those continuing to smoke appeared to be more likely to seek help for bone, joint or muscle conditions. Although strong conclusions cannot be drawn due to the small sample size, this could reflect improvements in circulation and inflammation following smoking cessation 1. This is a particularly novel finding and provides further support for the argument that, relative to continuing smokers, recent ex-smokers do not burden our health-care services. Their higher rate of dietician appointments at 6 months, relative to other types of appointments, could be due to recent ex-smokers attempting to adopt healthier life-styles generally and/or due to the weight gain which usually occurs following smoking cessation 16.

These findings have implications for cost–benefit analyses in that that there is no reason to include additional costs associated with smoking cessation as a consequence of increased health-care use. Smoking cessation is clearly beneficial in reducing the risk of morbidity and mortality, and consequently in reducing the economic burden on our health-care services in the long term 2,3. If smoking were to be eradicated this would save the National Health Service (NHS) in England £5.2 billion 4, with even larger health-care savings in other countries 5. Although there are currently significant competing demands for scarce health-care resources 23, emphasis upon and funding of evidence-based tobacco control interventions and policies is not undermined by any evidence for short-term health-care cost increases following cessation. In fact, smoking cessation interventions are among the most cost-effective ways of improving public health, and have been described as the gold standard to which other health promotion programmes should be compared 24.

This study has a number of advantages over previous studies: its ability to control for multiple confounding variables; its use of multiple health-care outcomes; and the use of cross-country data. The study also has a number of limitations. First, as data were collected online all measures involved self-report, although self-reported data have been shown to be valid indicators of smoking status 25. Secondly, due to the sample size we were unable to determine whether or not differences existed in the types of conditions for which individuals sought help. This would be a useful inclusion in future studies if enough smokers could be recruited. Thirdly, a number of smokers were lost to follow-up. This potential loss to follow-up was anticipated in the study design, with 2000 smokers recruited on the basis of a 50% dropout in order to attain adequate power. Differences between those followed-up and not followed-up were small, and previous studies have shown that the ATTEMPT cohort have characteristics similar to those found in national surveys of smokers 18. Finally, the medical conditions adjusted for in the current study were not exhaustive of all those that might be affected by smoking cessation. There are also issues with some of the classification systems; for example, ‘nervous system condition’ includes illnesses which vary to differing extents in their biological and psychological underpinnings.

## Conclusion

In a multi-national cohort of smokers, the current study found no substantial short-term change in health-care use, as measured by ER visits, hospitalization and appointments, when smokers stop.

### Declaration of interests

L.S. has received an honorarium for a talk and travel expenses from a pharmaceutical company making smoking cessation products. R.W. undertakes research and consultancy for developers and manufacturers of smoking cessation treatments such as nicotine replacement products. E.B. has received conference funding from Pfizer. S.J.C. served as a member of the scientific advisory board for this study in 2004 and in that capacity received reimbursement for travel and consultation.

## Supporting Information

Table S1 Baseline sample characteristics by follow-up status.
