# Supplementary material for: Association between smoking cessation and short-term health-care use: results from an international prospective cohort study (ATTEMPT)
Source: Addiction. 2013 Aug 14;108(11):1979–88. doi: 10.1111/add.12281 (PMC4282030; doi:10.1111/add.12281)
Supplement: Table S1 — Baseline sample characteristics by follow-up status. [file add0108-1979-SD1.docx]

**Supplementary material: Comparison of the final sample with those who dropped out**

Those who did not complete the three month follow-up questionnaire differed in terms socio- Those followed-up were significantly more likely to be married (X^2^=7.42, df 1, *p*=0.006), were less likely to be female (X^2^=14.81, df 1, *p*<0.001), and were less likely to report that they had been diagnosed with a skin condition (X^2^=4.73, df 1, *p*=0.030). demographic and physical characteristics. The country of residence of those followed-up also differed to those not followed-up (X^2^=37.26, df 4, *p*<0.001; they were less likely to be from the UK (*p*<0.001). Those who did not complete the six month follow-up questionnaire also differed on a few socio-demographic and physical characteristics. Those followed-up were less likely to be female (X^2^=11.99, df 1, *p*=0.001) and to report a nervous system condition (X^2^=4.78, df 1, *p*=0.029) and/or respiratory condition (X^2^=8.56, df 1, *p*=0.003), were more likely to be married (X^2^=10.14, df 1, *p*=0.001) and on average had a lower FTND score (t=-2.92, df 3563, *p*=0.003) and were of an older age (t=-2.09, df 3245.68, *p*=0.037). They also differed in terms of country of residence (X^2^=66.21, df 4, *p*<0.001); they were more likely to be from the US and less likely to be from the UK (*p*<0.001). Finally, those who did not complete the 12 month follow-up questionnaire were less likely to be female (X^2^=9.55, df 1, *p*=0.002) and were more likely to be married (X^2^=16.64, df 1, *p*<0.001). They were also less likely to report a nervous system condition (X^2^=4.89, df 1, *p*=0.027) and/or respiratory condition (X^2^=6.18, df 1, *p*=0.013), were of a younger age on average (t=3.54, df 3430.53, *p*<0.001) and had a lower BMI (t=3.54, df 3643, *p*<0.001). They also differed in terms of country of residence relative to those followed-up (X^2^=119.20, df 4, *p*<0.001); they were more likely to be from the US and less likely to be from the UK (*p*<0.001) (see supplementary Table 1).

Supplementary Table 1: Baseline sample characteristics by follow-up status

|  |  | **Followed up at 3 months** | | **Followed up at 6 months** | | **Followed up at 12 months** | |
| --- | --- | --- | --- | --- | --- | --- | --- |
|  | **Entire sample (n=3645)** | **Yes (n=2485)** | **No (n=1160)** | **Yes (n=2175)** | **No (n=1470)** | **Yes (n=1640)** | **No (n=2005)** |
| **Socio-demographic characteristics** |  |  |  |  |  |  |  |
| Mean (SD) Age | 45.8 (7.3) | 45.9 (7.38) | 45.6 (7.10) | 46.0 (7.42) | 45.5 (7.09)* | 46.3 (7.47) | 45.4 (7.12)*** |
| % (n) Women | 48.0 (1750) | 45.8 (1139) | 52.7 (611)*** | 45.7 (993) | 51.5 (757)*** | 45.2 (741) | 50.3 (1009)** |
| % (n) Married^~^ | 47.4 (1729) | 49.2 (1217) | 44.2 (512)** | 49.7 (1079) | 44.4 (650)*** | 51.3 (839) | 44.5 (890)*** |
| % (n) Employed^#^ | 72.2 (2632) | 72.4 (1799) | 71.8 (833) | 73.0 (1587) | 71.1 (1045) | 73.5 (1205) | 71.2 (1427) |
| % (n) Country  US  Canada  UK  France  Spain | 19.9 (725)  2.8 (102)  33.0 (1203)  33.2 (1209)  11.1 (406) | 19.9 (495)  2.9 (72)  30.0 (745)  35.1 (872)  12.1 (301) | 19.8 (230)  2.6 (30)  39.5 (458)***  29.1 (337)  9.1 (105) | 22.0 (479)  3.1 (68)  28.0 (609)  34.4 (748)  12.5 (271) | 16.7 (246)***  2.3 (34)  40.4 (594)***  31.4 (461)  9.2 (135) | 26.6 (437)  3.5 (57)  26.6 (436)  30.9 (507)  12.4 (203) | 14.4 (288)***  2.2 (45)  38.3 (767)***  35.0 (702)  10.1 (203) |
| % (n) White ethnicity | 93.3 (3400) | 93.7 (2328) | 92.4 (1072) | 93.2 (2027) | 93.4 (1373) | 92.7 (1521) | 93.7 (1879) |
| **Cigarette smoking characteristics** |  |  |  |  |  |  |  |
| Mean (SD) FTND Score ¥ | 4.7 (2.4) | 4.7 (2.41) | 4.8 (2.30) | 4.6 (2.37) | 4.9 (2.38)** | 4.7 (2.40) | 4.7 (2.36) |
| **Physical / mental health characteristics** |  |  |  |  |  |  |  |
| Mean (SD) Body Mass Index | 26.8 (5.5) | 26.8 (5.57) | 26.7 (5.34) | 26.9 (5.57) | 26.6 (5.38) | 27.1 (5.45) | 26.5 (5.52)*** |
| % (n) Heart or circulation condition^a ^^  % (n) Respiratory condition^b ^^  % (n) Endocrine, hormone or metabolic disorder^c ^^  % (n) Digestive condition^d ^^  % (n) Nervous system condition^e ^^  % (n) Cancer^f ^^  % (n) Ear, nose or throat condition^g ^^  % (n) Bone, joint or muscle condition^h ^^  % (n) Skin condition^i ^^  % (n) Reproductive or urinary condition^j ^^  % (n) Other ^^^ | 38.0 (1385)  25.5 (928)  20.7 (754)  18.0 (657)  39.5 (1439)  4.1 (148)  35.0 (1276)  32.1 (1170)  19.1 (697)  16.4 (596)  13.1 (477) | 38.1 (943)  24.7 (611)  20.2 (500)  17.6 (434)  38.7 (957)  4.2 (105)  34.7 (857)  32.3 (795)  18.2 (451)  16.0 (395)  12.8 (316) | 38.3 (422)  27.4 (317)  22.0 (254)  19.3 (223)  41.7 (482)  3.7 (43)  36.3 (419)  32.5 (375)  21.3 (246)*  17.4 (201)  13.9 (161) | 38.8 (844)  23.7 (516)  20.8 (452)  17.1 (373)  38.0 (827)  4.2 (91)  34.3 (746)  32.1 (699)  18.9 (410)  16.0 (348)  12.8 (279) | 36.8 (541)  28.0 (412)**  20.5 (302)  19.3 (284)  41.6 (612)*  3.9 (57)  36.1 (530)  32.0 (471)  19.5 (287)  16.9 (248)  13.5 (198) | 37.7 (619)  23.5 (385)  21.2 (347)  17.3 (283)  37.5 (615)  3.7 (61)  35.2 (577)  32.4 (532)  18.3 (300)  16.6 (273)  13.0 (214) | 38.2 (766)  27.1 (543)**  20.3 (407)  18.7 (374)  41.1 (824)*  4.3 (87)  34.9 (699)  31.8 (638)  19.8 (397)  16.1 (323)  13.1 (263) |

^a^ includes (1) angina pectoris (2) venous insufficiency (3) peripheral artery disease (4) heart attack, heart disease or other heart condition (5) hypertension (6) high cholesterol or abnormal triglycerides) (7) stroke; ^b^ includes (1) Asthma (2) Acute bronchitis (3) COPD (4) Other respiratory condition; ^c^ includes (1) Diabetes (2) Obesity; ^d^ includes (1) Irritable bowel syndrome, Crohn’s disease or ulcerative colitis (2) Stomach, duodenal or peptic ulcer (3) Hepatitis or cirrhosis; ^e^ includes (1) Depression (2) Anxiety (3) Multiple Sclerosis; ^f^ includes (1) Cancer of the lung, throat, mouth, pancreas, bladder, uterus, oesophagus or kidney (2) Cancer of some other type; ^g^ includes (1) hay fever, allergic rhinitis, seasonal allergies, sinusitis (2) Chronic sinus infection (3) Chronic laryngitis or pharyngitis (4) Gum disease; ^h^ includes (1) Osteoporosis (2) Arthritis, gout, lupus or fibromyalgia (3) Chronic back pain (4) Chronic joint pain (5) Chronic muscle pain (6) other chronic pain; ^I^ includes (1) wrinkles or skin aging (2) Other skin condition; ^j^ includes (1) Sexual dysfunction (2) Menopause (3) Kidney or renal failure; ^~^11 missing; ^#^9 missing; ¥ 49 missing;^^^18 missing; *p<0.05, **p<0.01, ***p<0.001.
